# A Rare Case of Metastasis to the Pancreas From a Primary Transitional-Cell Urinary Bladder Carcinoma

**DOI:** 10.7759/cureus.31580

**Published:** 2022-11-16

**Authors:** Labdhi H Rathod, Suraiya Ferdous, Mayur B Wanjari

**Affiliations:** 1 Department of Medicine, Jawaharlal Nehru Medical College, Datta Meghe Institute of Medical Sciences (Deemed to be University), Wardha, IND; 2 Department of Physiology, Jawaharlal Nehru Medical College, Datta Meghe Institute of Medical Sciences (Deemed to be University), Wardha, IND; 3 Research, Jawaharlal Nehru Medical College, Datta Meghe Institute of Medical Sciences (Deemed to be University), Wardha, IND

**Keywords:** endoscopic retrograde cholangiopancreatography, computed tomography, yellowish discoloration, obstructive jaundice, pancreatic mass

## Abstract

Pancreatic cancer (PC) occurs when changes (mutations) in the pancreas cells lead them to multiply out of control. A mass of tissue can result. Sometimes, this mass is benign (not cancerous). In PC, however, the mass is malignant (cancerous). A 79-year-old male presents to the emergency department with complaints of yellow discoloration of the eyes and body as well as itching all over the body. On general examination, his vitals were normal. Laboratory investigations showed raised levels of bilirubin and hepatic enzymes. The CT abdomen study revealed abnormal enhancing soft tissue density in the pancreatic head region obstructing the pancreatic duct and common bile duct leading to its proximal gross with upstream dilatation. The lesion described lesion in the pancreatic head region is of a malignant neoplastic etiology and needed to be evaluated with endoscopic retrograde cholangiopancreatography (ERCP), biopsy, and histopathology correlation. Associated pancreatic parenchymal atrophy was also seen along with gross dilatation of the extrahepatic and intrahepatic biliary system. There were no associated enlarged draining lymph nodes and minimal free fluid was seen in the peritoneal cavity. Following the deployment of a stent in the common bile duct, the patient was discharged. The patient prognosis was good and the patient was scheduled for routine blood work after four weeks to assess bilirubin levels and hepatic enzymes.

## Introduction

Obstructive jaundice is the most prevalent sign of pancreatic head malignancy. Surgical resection is the gold standard treatment option for individuals with a resectable tumor and no radiologic signs of spread. Preoperative biliary drainage due to significantly elevated bilirubin was developed to enhance the postoperative result since it is believed that surgery in patients with jaundice increases the risk of postoperative complications [1]. Pancreatic cancer (PC) is an aggressive malignancy with poor survival rates at advanced stages. Therefore, early identification is thought to be the best strategy for improving survival. To provide consensus criteria for the surveillance of people with a familial or hereditary risk of developing PC, the International Cancer of the Pancreas screening (CAPS) Consortium was initially convened in Baltimore in 2011 [2].

People with a long family history of PC or who are genetically predisposed to it are more likely to acquire the disease over several years. Clinicians should pick patients most likely to benefit from pancreas monitoring, talk to them about its advantages and disadvantages, and treat patients with lesions found by surveillance as best they can [2].

The fact that PC is rarely identified at an early stage is one of the reasons influencing the patient's poor prognosis. However, it was challenging to detect PC since several variables, including gender, age, main pancreatic dilatation, tumor marker, and the blood amylase level at admission, were not substantially linked to the disease. Only the discovery of a pancreatic mass allowed for the early identification of PC, even though 41.7% of those affected by PC had their mass recognized at the first sign of acute pancreatitis. PC can cause upstream PD dilation and post-obstructive pancreatitis. A key indicator of PC is the main pancreatic dilatation [3].

## Case presentation

A 79-year-old male presented to the emergency department with a complaint of yellowish discoloration of skin and eyes, yellow discoloration of urine and stools for 15 days, and pruritus for two months. The patient had noticed a gradual decrease in appetite for one month. The patient had no episodes of abdominal pain, nausea, vomiting, fever, cold, or cough in the past two months and no history of alcohol and tobacco. He had a history of diabetes mellitus type II and hypertension for three years and was taking medication for it. A year prior, the patient had transitional cell carcinoma of the bladder and had undergone nephroureterectomy for the resection of carcinoma. Biopsy was performed in which the section from the resected specimen of bladder tumor showed histopathological features of transitional cell carcinoma (urothelial malignancy-high grade) and section from deep muscle biopsy showed remarkable fibromuscular tissue and focal collection of chronic non specific inflammatory infiltrate on histopathology. Malignant epithelial cells were seen in histopathology suggestive of infiltration.

**Figure 1:**
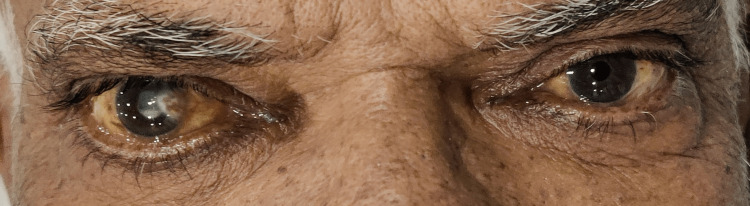
Yellowish discoloration of the eyes

**Figure 2:**
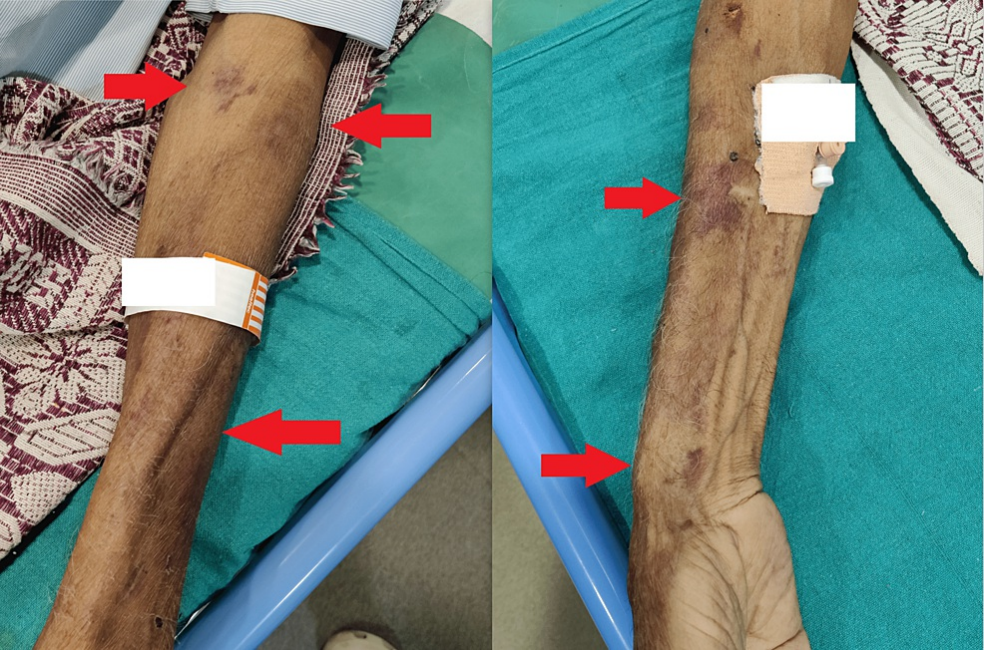
Lesions of the skin (red arrows)

**Figure 3:**
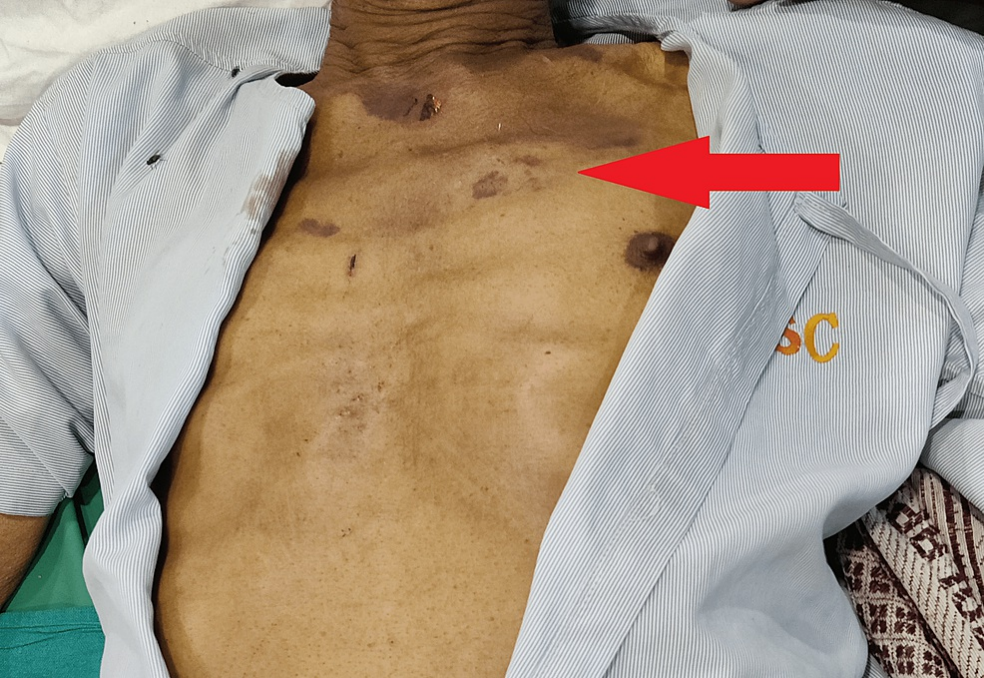
Lesions on the chest (red arrow) and paleness

On general examination and laboratory investigations, the patient’s vitals were normal but his skin and eyes were yellowish with the presence of lesions on the hands and chest (Figures 1-3). Elevated bilirubin levels and hepatic enzymes were discovered in laboratory investigation (Table 1).

**Table 1:** Liver function rest reports SGPT: Glutamic-pyruvic transaminase; SGOT: Glutamic-oxalacetic transaminase

Test	Results	Units	Reference Range
Bilirubin - Total	12.6	mg%	0.3-1.2
Bilirubin - Direct	9.27	mg%	0.0-0.3
Bilirubin - Indirect	3.33	mg%	0.1-1.0
SGPT	78	IU/L	5-40
SGOT	102	IU/L	5-40
Alkaline Phosphatase	382	IU/L	0-270
Total Protein	6.76	gm/dl	6-8
Albumin	3.55	gm/dl	3.5-5
Globulin	3.21	gm/dl	2.3-3.5

**Figure 4:**
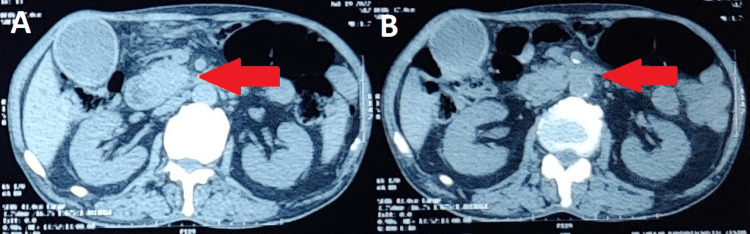
Different views (A and B) of CT scan showing a soft tissue density lesion in the head of the pancreas

On performing a CT scan of the abdomen and pelvis, an abnormal enhancing soft tissue density lesion in the head of the pancreas was observed, measuring approximately 25 × 22 mm in maximum cross-sectional dimension. The lesion obstructed the common bile duct and the pancreatic duct, leading to its dilatation. There was a gross dilatation of the pancreatic duct with significant atrophy of the pancreatic parenchyma surrounding the dilated duct (Figure 4).

**Figure 5:**
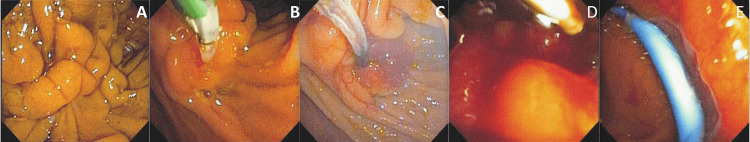
A) Ampullary mass; B-E) Placement of the plastic stent

The pancreatic duct measures 13 mm in maximum diameter in the head region. The lesion is in close proximity with the posterior aspect of the superior mesenteric vein with the maintained plane of separation in between or just abutting the posterior aspect of the superior mesenteric vein. Its plane of separation with the superior mesenteric artery is also very well maintained. On the right side, it is in close proximity to the distal part of the second part of the duodenum and the proximal part of the third part of the duodenum with no obvious signs of infiltration. Posteriorly, its plane of separation with the inferior vena cava is well maintained. The patient's prognosis was good after endoscopic retrograde cholangiopancreatography (ERCP) and reduce clinical manifestation (Figure 5).

## Discussion

Obstructive jaundice is a prevailing condition all over the world. Its common cause includes neoplasms of the pancreas, gallbladder, biliary system, or the ampulla of Vater, choledocholithiasis, and chronic pancreatitis [4]. Due to PC's high prevalence and increased mortality rate, safe, highly sensitive and repeatable methods for diagnosing pancreatic mass lesions should be practiced. Serum tumor markers, ERCP, and endoscopic ultrasound-guided fine needle aspiration (EUS-FNA) are currently used in addition to radiological imaging. EUS-FNA has been the standard method for diagnosing pancreatic mass lesions. Staging PC is of prime importance to regulate the resectability of the tumor. About 10-15% of people that suffer from PC are eligible for surgical resection to prevent the risk of spreading of tumor to other parts of the body [5].

ERCP is often used to assess the patient with obstructive jaundice. ERCP is a more precise measure to detect lesions which are difficult to detect in CT or transcutaneous sonography [6]. ERCP is a method used to inspect tumors of the pancreatico-biliary junction. 85% of these tumors are pancreatic, 6% originate in the distal common bile duct and 4.5% are ampullary or duodenal carcinomas. ERCP grants the depiction of the biliary tract and the bile duct [7].

The main pancreatic duct (MPD) can also be examined with ERCP to check whether the tumor is obstructing it. During initial hospitalization, ERCP is not performed due to the high-risk factor of post-ERCP pancreatitis [3]. The only potentially viable treatment for PC is surgical resection. However, over 80% of tumors cannot be removed at diagnosis. Chemotherapy is the preferred treatment for people with advanced PC, even though the regimen's severe side effects make them inappropriate for patients with low-performance status. Therefore, it is crucial to identify PC early to offer patients the best possible course of treatment [8]. The outcomes for surgical removal of pancreatic lesions have improved recently. The use of endoprosthesis brought about the improvement in palliation. Despite the disappointing outcomes, we must actively pursue more precise diagnostic and therapeutic approaches [7].

## Conclusions

In conclusion, ERCP is an appropriate and precise technique for diagnosing the extrahepatic biliary tree obstruction. Our results suggest that the common cause of obstructive jaundice was the development of a soft tissue density lesion in the head of the pancreas, which led to PC diagnosis. The degree of precision in this technique is quite comparable to other techniques like CT, ultrasonography, etc., to gauge the frequency of serious complications. We believe that the surgical care of obstructive jaundice would be improved by using ERCP to identify the condition's etiology.
